# COVID-19 Pandemic Impact on Care for Stroke in Australia: Emerging Evidence From the Australian Stroke Clinical Registry

**DOI:** 10.3389/fneur.2021.621495

**Published:** 2021-02-26

**Authors:** Dominique A. Cadilhac, Joosup Kim, Emma K. Tod, Julie L. Morrison, Sibilah J. Breen, Katherine Jaques, Rohan Grimley, Brett Jones, Geoffrey C. Cloud, Timothy Kleinig, Susan Hillier, Helen Castley, Richard I. Lindley, Natasha A. Lannin, Sandy Middleton, Bernard Yan, Kelvin Hill, Benjamin B. Clissold, Peter J. Mitchell, Craig S. Anderson, Steven G. Faux, Bruce C. V. Campbell, The AuSCR COVID-19 Reporting Consortium Group

**Affiliations:** ^1^Stroke and Ageing Research School of Clinical Sciences at Monash Health, Monash University, Clayton, VIC, Australia; ^2^Stroke Division, The Florey Institute of Neuroscience and Mental Health, Heidelberg, VIC, Australia; ^3^Statewide Stroke Clinical Network, Healthcare Improvement Unit, Clinical Excellence, Queensland Health, Brisbane, QLD, Australia; ^4^Canberra Hospital, Canberra, ACT, Australia; ^5^Department of Neuroscience, Central Clinical School, Monash University, Clayton, VIC, Australia; ^6^Alfred Health, Prahran, VIC, Australia; ^7^Royal Adelaide Hospital, Adelaide, SA, Australia; ^8^Division of Health Sciences, University of South Australia, Adelaide, SA, Australia; ^9^Royal Hobart Hospital, Hobart, TAS, Australia; ^10^Westmead Clinical School, University of Sydney, Darlington, NSW, Australia; ^11^Nursing Research Institute, Australian Catholic University, Sydney, NSW, Australia; ^12^St Vincent's Hospital Sydney, Darlinghurst, NSW, Australia; ^13^Department of Radiology, The Royal Melbourne Hospital, University of Melbourne, Parkville, VIC, Australia; ^14^Stroke Foundation, Melbourne, VIC, Australia; ^15^Monash Health, Clayton, VIC, Australia; ^16^Faculty of Medicine, University of New South Wales, Sydney, NSW, Australia; ^17^The George Institute for Global Health, Camperdown, NSW, Australia; ^18^University of New South Wales, Kensington, NSW, Australia

**Keywords:** stroke, COVID-19, healthcare quality, survey, clinical registry

## Abstract

We present information on acute stroke care for the first wave of the COVID-19 pandemic in Australia using data from the Australian Stroke Clinical Registry (AuSCR). The first case of COVID-19 in Australia was recorded in late January 2020 and national restrictions to control the virus commenced in March. To account for seasonal effects of stroke admissions, patient-level data from the registry from January to June 2020 were compared to the same period in 2019 (historical-control) from 61 public hospitals. We compared periods using descriptive statistics and performed interrupted time series analyses. Perceptions of stroke clinicians were obtained from 53/72 (74%) hospitals participating in the AuSCR (80% nurses) *via* a voluntary, electronic feedback survey. Survey data were summarized to provide contextual information for the registry-based analysis. Data from the registry covered locations that had 91% of Australian COVID-19 cases to the end of June 2020. For the historical-control period, 9,308 episodes of care were compared with the pandemic period (8,992 episodes). Patient characteristics were similar for each cohort (median age: 75 years; 56% male; ischemic stroke 69%). Treatment in stroke units decreased progressively during the pandemic period (control: 76% pandemic: 70%, *p* < 0.001). Clinical staff reported fewer resources available for stroke including 10% reporting reduced stroke unit beds. Several time-based metrics were unchanged whereas door-to-needle times were longer during the peak pandemic period (March-April, 2020; 82 min, control: 74 min, *p* = 0.012). Our data emphasize the need to maintain appropriate acute stroke care during times of national emergency such as pandemic management.

## Introduction

Stroke is a time critical emergency and is a leading cause of death and disability. Better outcomes are achievable with early presentation to hospital and treatment in a specialized stroke unit (1). Worldwide, there have been reports that the Coronavirus Disease 2019 (COVID-19) pandemic has impacted presentations to hospital and the ability to provide the same standard of acute stroke care as prior to the pandemic. During this time, national organizations for stroke have issued statements to encourage people with suspected stroke to avoid delays in seeking medical attention and to promote and preserve best practice management in hospitals (2).

The impacts on emergency department presentations and admissions to hospitals for acute stroke during the COVID-19 pandemic have been reported (3–13). The steepest declines in presentations have been noted to immediately follow implementation of lockdown or stay at home orders (10–12). Several authors have reported a change in symptom severity of people presenting with stroke or transient ischemic attack (TIA). In some studies, presentations of minor strokes and TIAs have reduced by 31–45% (3, 4, 7, 10, 11, 13). The impacts on patient arrival times to hospital (3, 6, 7, 12–14) and the delivery of stroke care that have been reported has differed between studies (4). Reported changes have included altered transportation protocols, changes to triage processes and stroke pathways, and fewer multidisciplinary team rounds (15, 16). Provision of reperfusion treatments and the timeliness of these have decreased or remained stable (3, 5, 6, 8, 12, 17, 18).

Within Australia, social distancing restrictions and community lockdowns commenced on March 24, 2020; and almost uniquely in the developed world, were followed by extremely low COVID-19 transmission rates. Guidance on the use of personal protective equipment at hospitals was regularly updated by the Australian Government. The first case of COVID-19 in Australia was recorded on January 25th and by June 30th 2020, there were 7,833 cases (307 per 1,000,000 population) and 104 deaths (19), with the majority of cases (69%) occurring in two Australian states (Supplementary Table 1). Since the experience in each country to COVID-19 and its impact on stroke is likely to differ, we provide this brief report to illustrate the unintended and indirect consequences from an Australian perspective.

## Aims

The aim of this paper is to describe the impact of the COVID-19 pandemic on stroke services from the perspective of hospitals participating in the Australian Stroke Clinical Registry (AuSCR) up until the end of June 2020.

## Methods

The impacts of the COVID-19 pandemic on stroke services were captured through our collaborative work on the AuSCR. The AuSCR is a clinical quality registry that was established in 2009 to monitor and improve the quality of stroke care in Australia. Participating hospitals capture data on patients admitted with acute stroke and TIAs. Public hospitals from six Australian states and territories contribute data to the AuSCR and it is funded primarily by state governments. The AuSCR captures a minimum dataset on patient characteristics, clinical indicators, and patient outcomes. The data for the analysis were extracted from AuSCR on the 17th September 2020. For further information see www.auscr.com.au.

### Study Design

Mixed methods design using (i) patient-level data from the registry and (ii) a survey of clinicians working in the hospitals that participate in the AuSCR.

To avoid the effect of season on stroke admissions, patient-level data from the registry from January to June 2020 were compared to data for the same period in 2019 prior to the pandemic (the historical control cohort for this analysis). We also compared the 2 months when we had the peak of cases in the first wave of COVID within Australia occurred between March and April (https://covid-19-au.com/, accessed 21 September 2020) to the historical, non-pandemic control period.

### Setting

Public hospitals from six Australian states and territories that contribute data to the AuSCR.

### Survey Design and Distribution

A voluntary, feedback survey was designed to capture the impacts of COVID-19 from the perspective of hospital clinicians working in on acute stroke care using a standardized approach. Several members of the AuSCR team (EKT, JLM, SJB, DAC) developed the set of 23 closed or open-ended questions based on informal feedback and information being circulated by the Stroke Foundation and World Stroke Organization. The questionnaire was reviewed and approved for distribution by the Chair of the Management (NAL) and Steering committees (SM). A copy of the survey is in the Supplementary Material.

The survey was distributed electronically to the AuSCR hospital mailing list and *via* the AuSCR Newsletter on the first of May 2020 and was left open until the 31st of May 2020. More than one response from each participating hospital was acceptable. Participation was voluntary.

### Data Analysis

Quantitative and qualitative methods of analyses were used as outlined below. Results of the survey were contrasted to the findings from the comparison of patient data from the registry to provide context.

#### Analysis of Patient Cohort Data From the Australian Stroke Clinical Registry

Hospitals contributing data between January and June 2019 (historical-control period) and between January and June 2020 (pandemic period) were included in a matched analysis. Patient characteristics (stroke numbers, type, severity, age) and quality of care indicators (treatment in a stroke unit, provision of reperfusion and time to reperfusion), and other process metrics (i.e., length of stay and discharge destination) were compared. Descriptive statistics were generated based on the type and distribution of the data. Differences between time periods were assessed using Chi^2^ and Kruskal-Wallis tests. In the case where a specific process of care (i.e., clinical indicator) for any individual hospital contained >30% missing data, these hospitals were excluded from the analysis of that specific process of care. Data on the provision of stroke unit care was excluded from one hospital contributing 86 cases between 1/1/2019 and 17/9/2020.

Data on all episodes between 1/1/2019 and 17/9/2020 at the hospitals included in the matched analysis were used in an interrupted time series (ITS) analyses. Data points are presented per week. For the ITS analyses related to reperfusion, predicted values were adjusted for the number of patients with ischemic stroke. All other ITS analyses were adjusted for the numbers of patients with different diagnoses (ischemic stroke, intracerebral hemorrhage, transient ischemic attack, and undetermined stroke). Trends were compared before and after 1/3/2020 (week 61 in the model), which coincided with the first COVID-19 related death in Australia. A State of Emergency was declared in Victoria on 16/3/2020 and there were nationwide restrictions imposed on 21/3/2020. Seasonality was considered by using a lag period of 53 weeks for correlations. The last 2 weeks of data were removed due to low case numbers entered into the AuSCR (Supplementary Figure 1).

#### Analysis of Survey From Clinicians That Participate in the Australian Stroke Clinical Registry

Descriptive statistics were compiled for closed questions and inductive thematic analysis for open-ended responses.

## Results

### Comparison of Patient-Level Registry Data

Sixty-two hospitals from five states (New South Wales, Victoria, Queensland, South Australia, and Tasmania) contributed patient data between January and June in both 2019 and 2020. We did not include data from a children's hospital, therefore the data used in the analysis was from 61 hospitals (Supplementary Table 1). Data from the registry covered locations that had 91% of Australian COVID-19 cases to the end of June 2020.

A sample of 9,308 pre-COVID 2019 AuSCR episodes were compared to a 2020 sample of 8,992 episodes (Supplementary Table 2). The demographic characteristics and stroke type distributions were similar for each cohort (median age: 75 years; 56% male; ischemic stroke 69%).

**Figure 1:**
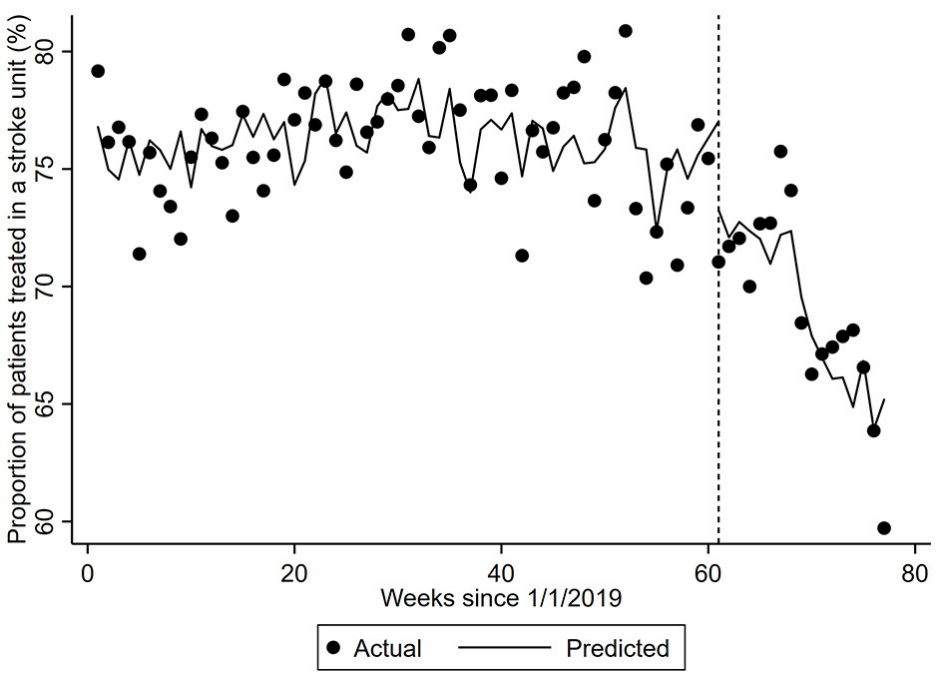
Proportion of patients treated in a stroke unit. The dots represent the proportion of patients provided treatment in a stroke unit per week. The predicted proportion is the expected proportion is adjusted for the number of patients with different diagnoses and the corresponding number from the previous year (seasonality). Vertical hatched line represents week 61 of the interrupted time-series model with trends compared before and after this date, which coincides with the first COVID-19 related death. Coefficient −0.2457 (95% CI −0.4216, −0.0697, *p*-value = 0.0069).

There were several changes in clinical processes over the first half of 2020 (Table 1). Compared to the pre-pandemic period, more patients arrived to hospital by ambulance in March–April 2020 (*p* < 0.001) and May–June (*p* = 0.001) and there was a shorter time between hospital arrival and brain scan (*p* < 0.001 and *p* = 0.041, respectively). The proportion of patients treated in a stroke unit in the first half of 2020 was significantly less than those in the control period (*p* < 0.001 for each period) and appeared to decline over the first half of 2020 (from 73 to 65%, *p* < 0.001). Using ITS analysis, the proportion of patients treated in a stroke unit decreased by 2.85% (95%CI −4.19, −1.51) in the week including 1/3/2020 followed by a continued decline of 0.25% per week (Figure 1).

**Table 1:** Comparison of patient characteristics and clinical care for the historical-control period and different stages of the pandemic in 2020.

	**2019**	**2020**					
	**January–June 2019 (control period)**	**January/February (pandemic period 0)**		**March/April (pandemic period 1)**		**May/June (pandemic period 2)**	
Number of episodes	9,308	3,180		3,017		2,795	
Number of patients	9,116	3,080		2,912		2,737	
	***n*** (%)^*^	***n*** (%)^*^	***p*****-value**^†^	***n*** (%)^*^	***p*****-value**^†^	***n*** (%)^*^	***p*****-value**^†^
Median age (IQR)	75 (64–83)	75 (64–83)	0.961	74 (64–83)	0.094	76 (66–84)	0.014
Male	5,102 (56)	1,767 (57)	0.373	1,670 (56)	0.810	1,516 (55)	0.229
Diagnosis			0.060		0.636		<0.001
Intracerebral hemorrhage	1,124 (12)	326 (10)		350 (12)		331 (13)	
Ischemic stroke	6,393 (69)	2,219 (71)		2,058 (70)		1,628 (65)	
Transient ischemic attack	1,521 (16)	502 (16)		468 (16)		505 (20)	
Undetermined stroke	201 (2)	71 (2)		55 (2)		58 (2)	
Ability to walk on admission	3,772 (43)	1,230 (44)	0.704	1,061 (42)	0.091	868 (41)	0.082
Arrival by ambulance	6,802 (77)	2,177 (77)	0.816	2,110 (81)	<0.001	1,840 (80)	0.001
Median minutes from onset to arrival (IQR)	337 (100–1,127)	364 (105–1,192)	0.317	340 (98–1,074)	0.622	344 (101–1,013)	0.673
Median minutes from door to brain scan (IQR)	57 (24–155)	57 (24–153)	0.768	48 (22–120)	<0.001	51 (24–139)	0.041
Thrombolysis if ischemic stroke	808 (13)	246 (11)	0.082	247 (12)	0.545	178 (11)	0.066
Median minutes from door to needle (IQR)	74 (54–101)	76 (56–104)	0.395	82 (59–116)	0.012	71 (52–103)	0.882
Door to needle time under 60 min	218 (27)	65 (26)	0.863	56 (23)	0.177	46 (26)	0.756
Endovascular thrombectomy if ischemic stroke	535 (18)	183 (20)	0.400	159 (18)	0.992	144 (20)	0.266
Median door to groin puncture (minutes IQR)	76 (32–122)	81 (31–126)	0.643	84 (38–123)	0.504	89 (48–122)	0.074
Treated in a stroke unit	7,059 (76)	2,307 (73)	<0.001	2,126 (70)	<0.001	1,822 (65)	<0.001

IQR, interquartile range;

* number and proportion of the number of episodes unless otherwise specified;

† *P-value is for the differences between pandemic periods in 2020 compared to historical control period January to June 2019*.

*Missing data: age 2.7%, sex 1.8%, diagnosis 2.8%, ability to walk on admission 11.9%, arrival by ambulance 9.3%, onset to arrival 14.2%, door to brain scan 9.8%, thrombolysis 2.6%, door to needle 11.1%, endovascular thrombectomy 3.1%, door to groin 12.7%, treated in a stroke unit 3.4%*.

**Figure 2:**
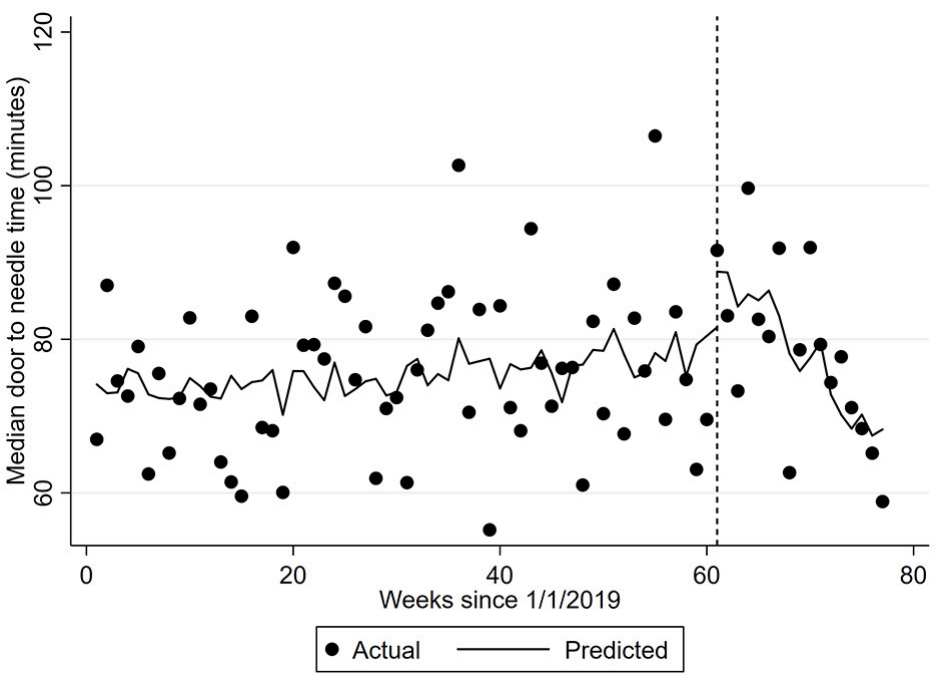
Median door-to-needle time. The dots represent the median door-to-needle per week. The predicted time is adjusted for the number of patients with ischemic stroke and the corresponding number from the previous year (seasonality). Vertical hatched line represents week 61 of the interrupted time-series model with trends compared before and after this date, which coincides with the first COVID-19 related death. Coefficient −2.0476 (95% CI −2.36, −1.73) *p* < 0.0001.

During the week including 1/3/2020, the proportion of patients with ischemic stroke provided thrombolysis increased by 1.09% (Table 2). There was a 0.03% per week decrease in thrombolysis provision per week prior to this point and a significantly greater 0.23% per week decrease (95% CI −0.35, −0.10) from the week including 1/3/2020. Median door-to-needle times increased by 0.10 (95% CI 0.03, 0.18) minutes per week prior to the 1/3/2020, followed by a 12.15 minute (95% CI 9.35, 14.95) increase during the week including 1/3/2020 (Figure 2). Median door-to-needle times decreased by 2.05 (95%CI −2.36, −1.73) minutes per week from the week including 1/3/2020. When data from Victoria and Queensland were explored using ITS analysis, given these two states of Australia provided 75% of the AuSCR data in this analysis and had 40% of the COVID-19 cases (Supplementary Table 1), the only notable difference was that Victoria had a more pronounced reduction in stroke unit care in the interruption week [Victoria ~6% drop, Queensland ~4% increase (Supplementary Table 3)].

**Table 2:** Comparison of discharge care and length of stay for the historical-control period and different stages of the pandemic in 2020.

	**2019**			**2020**			
	**January–June 2019 (control period)**	**January/February (pandemic period 0)**	***p*-value^†^**	**March/April (pandemic period 1)**	***p*-value^†^**	**May/June (pandemic period 2)**	
Median length of stay (IQR)	4 (2–7)	4 (2–7)	0.001	3 (2–6)	<0.001	4 (2–7)	<0.001
Discharged home	4,593 (50)	1,602 (52)	0.062	1,430 (49)	0.394	1,193 (49)	0.231
Discharged to inpatient rehabilitation	2,027 (22)	619 (20)	0.020	679 (23)	0.160	558 (23)	0.468
Provided a discharge care plan if discharged to the community	3,127 (66)	1,127 (68)	0.052	940 (64)	0.412	810 (66)	0.756
Discharged with an antihypertensive medication	5,893 (74)	1,892 (70)	<0.001	1,751 (69)	<0.001	1,435 (62)	<0.001
Discharged with an antithrombotic medication (excludes ICH)	6,704 (91)	2,229 (87)	<0.001	2,010 (85)	<0.001	1,605 (75)	<0.001
Discharged with a lipid-lowering medication (excludes ICH)	5,674 (77)	1,954 (77)	0.669	1,754 (74)	0.001	1,369 (64)	<0.001

† *P-value is for the differences between pandemic periods in 2020 compared to historical control period January–June 2019; IQR, interquartile range; ICH, intracerebral hemorrhage*. *Missing data: length of stay 3.1%, discharge destination 4.2%, provided a discharge care plan 6.7%, discharged with an antihypertensive medication 11.4%, discharged with an antithrombotic medication 7.7%, discharged with a lipid-lowering medication 7.9%*.

Fewer patients were discharged to rehabilitation in January–February 2020 (*p* = 0.020) but this improved to pre-pandemic levels from March onwards (Table 3). The median length of stay was 3 days between March and April 2020, lower than a median of 4 days in other months in the first half of 2020 and significantly shorter than pre-pandemic period (*p* < 0.001). There was evidence of fewer patients receiving their secondary prevention medications at time of discharge (Table 3).

**Table 3:** Changes in the provision of acute stroke care assessed using interrupted time series analysis.

	**Trend before 1/3/2020**	Change during the week of 1/3/2020 (interruption week)^*^	**Trend from 1/3/2020**
Proportion provided thrombolysis if an ischemic stroke	−0.03 (−0.05, −0.02)	1.09 (0.05, 2.14)	−0.23 (−0.35, −0.10)
Median door to needle time (minutes)	0.10 (0.03, 0.18)	12.15 (9.35, 14.95)	−2.05 (−2.36, −1.73)
Proportion treated in a stroke unit	−0.01 (−0.04, 0.02)	−2.85 (−4.19, −1.51)	−0.25 (−0.42, −0.07)

*Coefficient and 95% confidence intervals presented*.

* *week 61 of the interrupted time-series model with trends compared before and after this date, which coincides with the first COVID-19 related death*.

### Survey Results

Responses were received from 53/72 (74%) hospitals participating in the AuSCR; 80% of respondents had a nursing background. About half reported reduced presentations, in particular for mild stroke and transient ischemic attacks (44%). Changes to patient flow and management in emergency departments were reported by 36%; 26% had their stroke unit relocated with 10% reporting reduced capacity, and 28% reporting that stroke service staff were redeployed to other hospital work. Staff described the impact on patients having less access to visitors creating reduced opportunities for support and education and information sharing with relatives by the stroke team. Communication about redeployment of services or patient flow policy changes was reported to be less than optimal at some hospitals. Delays to time-critical stroke treatment were identified due to COVID-19 screening/triage processes, and nursing staff ratios and the interdisciplinary team skill base were adversely impacted.

## Discussion

Despite Australia's relatively low rates of COVID-19 in the population [4.5 times fewer cases per million than the global average (20)], we have demonstrated that stroke care nationally was negatively impacted by the pandemic. This may have been brought on by the need to prepare for a potential influx of infected cases needing hospital management and additional screening processes for triage in emergency departments. In our study, significantly fewer patients had access to stroke unit care at all three post-pandemic timepoints analyzed, an important finding given that patients treated in a stroke unit are more likely to be alive, independent, and living at home 1 year after the stroke than if cared for in other settings (1, 21). Length of stay significantly decreased and fewer patients were discharged with secondary stroke prevention medications across the pandemic timepoints. Other care processes such as discharge to rehabilitation and door-to-needle times under 60 minutes for thrombolysis quickly returned to pre-pandemic levels. Interestingly, an increased proportion of strokes arrived by ambulance in the post-pandemic period, which coincided with a concomitant decrease in the median time to brain scan following arrival. There was little evidence to indicate that one region fared worse than others during this first wave of the pandemic.

Care in organized, dedicated stroke units by expert, interdisciplinary clinicians is the hallmark of best-practice stroke care that is universally applicable to all patients with stroke (21). Our major findings were that stroke unit access decreased, fewer patients were discharged to inpatient rehabilitation in the early phases of the pandemic, patients were being discharged earlier, and discharged without secondary prevention medications more often than the pre-pandemic period. This may have occurred due to resource redistribution between units, demand for hospital beds or clinician perception of increased risk of patients contracting COVID-19 in hospitals. Our quantitative findings mirror the feedback about service impacts reported by the stroke clinicians. These findings are concerning, since without the care provided in dedicated stroke units, patients experience more complications, disability and mortality (21–23). Disaster planning should incorporate the necessity of maintaining stroke services so that access to evidence-based care processes, such as stroke unit access, are sustained and the “collateral damage” of disaster responses is limited.

Interestingly, although clinicians reported that milder strokes were not presenting to hospital, we were unable to substantiate this perception of a clinically meaningful difference in severity based on the ability to walk on admission or the proportion admitted with final diagnosis of TIA. Evidence around symptom onset to arrival time to hospital has been mixed with reports of maintenance of usual onset to arrival time or delays, particularly with the proportion of ischemic strokes arriving within 4.5 h of onset (3, 6, 7, 12–14). In our study we did not detect a change in median arrival times to hospital from symptom onset. However, more patients used ambulance services. We have described the impact of the COVID-19 pandemic on stroke services from the perspective of hospitals participating in the AuSCR up until the end of June 2020. Although most jurisdictions in Australia were successful in containing the COVID-19 pandemic by the end of April 2020, community and hospital responses led to significant differences in stroke system organization and stroke care. Here we have provided an analysis of the first 6 months of the pandemic prior to the “second wave” of COVID-19 that was limited to Victoria and led to a large number of community transmissions with many health workers being infected (24).

Stroke symptom recognition, rapid diagnosis and subsequent treatment requires streamlined management across multiple hospital departments that is guided by an interdisciplinary team (25). The reported changes to the delivery of stroke services from different countries have not been uniform. While delivery of care in some hospitals and systems has not been significantly restructured, others have experienced redeployment of specialist staff and repurposing of dedicated stroke beds to meet the demands of increased patients requiring treatment for COVID-19 (4, 26, 27). Changes have included the necessity of establishing different triaging protocols for presentations with respiratory symptoms or other signs of COVID-19, altered transportation protocols, changes to stroke pathways and fewer multidisciplinary team rounds (15, 16).

Authors of studies conducted in other countries have reported changes to emergency department workflows associated with the COVID-19 pandemic whereby prevention strategies may delay referrals to the stroke team, and additional brain scanner decontamination processes have also created delays (8, 25, 27, 28). Treatment delays for those eligible for reperfusion therapies lead to worse outcomes including greater disability (29, 30). Our results are reassuring in that many time-based metrics were not negatively impacted. In our cohort, it was found that door-to-scan times were faster during the pandemic period compared to the control period, which may reflect decreased workload in the emergency department at the time, or the increased proportion of patients arriving by ambulance. Although door-to-needle times initially rose by 12.15 (95% CI 9.35, 14.95) minutes with commencement of the pandemic period, these subsequently declined progressively thereafter with evidence of returning to pre-pandemic trends after April. A trend for increased door-to-groin puncture times did not reach significance. Internationally, there have been reports of reduced total numbers of thrombolysis and endovascular thrombectomy procedures due to fewer presentation volumes which was not reflected in these Australian data. Rates of provision of reperfusion therapies among patient with ischemic strokes has been relatively stable (3, 5, 6, 8, 17, 18) both in this study and elsewhere, along with associated time metrics (5, 6, 12, 31).

The strengths of our study include the standardized data from the AuSCR on 18,300 stroke and TIA admissions to the majority of participating hospitals (*n* = 61), and obtaining clinician perspectives prior to the analysis. Further we used a historical control period matched to the pandemic period including comparisons with different phases of the first national wave to account for potential differences in stroke admission related to season. We also performed interrupted time series analysis to comprehensively understand the influence of trends in our prospectively collected patient data adjusting for seasonality and changes in the distribution of diagnosis. The analysis of the survey data was undertaken by different authors (EKT, DAC) to the author who analyzed the AuSCR data (JK). A limitation is the potentially incomplete data for 2020, and the potential for selection bias if not all AuSCR cases were captured because of constrained resources in hospitals to collect data. However, given that the number of 2020 episodes is similar to those collected in the 2019 pre-pandemic period, we are reasonably confident that 2020 dataset is a representative sample from the time of data extraction and analysis. Further, we were unable to report on whether any of the patients in our sample experienced stroke as a result of contracting COVID-19. There is emerging evidence that COVID-19 exhibits neurotropic properties and causes neurological diseases (32) and it is estimated that 1.5% of patients with emergency department visits or hospitalizations with COVID-19 have experienced ischemic stroke (33).

Future data from the registry will enable analysis of the long-term outcomes for patients with stroke who received care in the pandemic period when compared with the pre-pandemic period, including mortality and self -reported morbidity at 90–180 days following admission. A larger second wave of COVID-19 also occurred in the state of Victoria from June 2020 onwards, and future analyses will focus on this time period and comparisons between different regions of Australia. Linkage of AuSCR data with other datasets, to include confirmed diagnoses of COVID-19 positive patients, will also broaden our understanding of the relationship between COVID-19 and stroke.

During disruptions such as a pandemic, efforts to ensure that people with suspected stroke are provided with rapid triage, acute diagnosis and reperfusion treatment where relevant, and are provided with secondary prevention medications when discharged are required. Access to specialized stroke unit care and rehabilitation should not be compromised. Such concerns have resulted in the Stroke Society of Australasia's appeal to hospital executives to protect the integrity of stroke services (34).

## Conclusions

We highlight the consequences of community and health system COVID-19 responses on hospital care for acute stroke. These impacts occurred despite the predicted hospital overload from COVID-19 patients being averted in Australia during the first half of 2020. The continued decreased access to specialized stroke units is of grave concern given treatment in a stroke unit improves outcomes. Future studies of longer-term outcomes following significant changes to access to stroke unit care, accompanied by decreased length of stay, will be crucial. It is imperative that solutions are identified to maintain appropriate acute stroke care during times of national emergency such as pandemic management. These might include alternate models of providing support to patients immediately after stroke and without comprising access to best-practice in-patient care. As Australia cycles out of the initial phase of the COVID-19 pandemic we will need to ensure there is resilience within the health system to similar events in the future.

## Data Availability Statement

The data that support the findings of this study are available from the Florey Institute of Neuroscience and Mental Health. Restrictions apply to the availability of these data, which were used under license for this study. Data are available from the corresponding author with the permission of the Florey Institute of Neuroscience and Mental Health acting on behalf of the AuSCR Consortium. Requests to access these datasets should be directed to admin@auscr.com.au.

## Ethics Statement

This study was approved by the Monash University Human Research Ethics Committee (project ID number 26558). Written informed consent for participation was not required for this study in accordance with the national legislation and the institutional requirements.

## Author Contributions

DAC and JK: study design, data analysis and interpretation, and drafting of the article. ET, JM, and SB: study design, data collection, interpretation of data, and drafting of the article. KJ, RG, BJ, GC, TK, HC, RL, BCl, PM, and BCa: data collection, interpretation of data, and critical revision of the article. SH, NL, SM, BY, KH, CA, and SF: interpretation and critical revision of the article. All authors provided final approval of the version to the article to be published.

## AuSCR COVID-19 Reporting Consortium Group

The following authors contributed to the interpretation of data, critical revision of the article, and/or collection of data.

Alaa Alghamry (The Prince Charles Hospital, Queensland, Australia)

Lauren Arthurson (Echuca Regional Health, Victoria, Australia)

Jonnel Boco (Caboolture, Queensland, Australia)

Melanie Brown (Goulburn Valley Health, Victoria, Australia)

Helen Brown (Princess Alexandra Hospital, Queensland, Australia)

Ernie Butler (Peninsula Health, Victoria, Australia)

Greg Cadigan (Queensland Health, Queensland, Australia)

Anna Clissold (South West Healthcare, Victoria, Australia)

Janell Cole (North West Regional Hospital, Tasmania, Australia)

Douglas Crompton (Northern Health, Victoria, Australia)

Vanessa Crosby (Albury Wodonga Health, Victoria & New South Wales, Australia)

Geoffrey Donnan (University of Melbourne, Parkville, Australia)

Angela Dos Santos (Alfred Health, Victoria, Australia)

Ramesh Durairaj (Cairns Hospital, Queensland, Australia)

Chris Ebersohn (Wimmera Base Hospital, Victoria, Australia)

Andrew Evans (Westmead Hospital, New South Wales, Australia)

Brett Forge (West Gippsland Hospital, Victoria, Australia)

Karen Fuller (Wollongong Hospital, New South Wales, Australia)

Nisal Gange (Toowoomba Hospital, Queensland, Australia)

Yash Gawarikar (Calvary Hospital, Australian Capital Territory, Australia)

Richard Geraghty (Redcliffe Hospital, Queensland, Australia)

Kate Jackson (Agency for Clinical Innovation, New South Wales, Australia)

Martin Jude (Wagga Wagga Hospital, New South Wales, Australia)

Salman Khan (Nepean Hospital, New South Wales, Australia)

Monique Kilkenny (Monash University, Victoria, Australia)

Thomas Kraemer (Ballarat Base Services, Victoria, Australia)

Alex Lau (Logan Hospital, Queensland, Australia)

Henry Ma (Monash Health, Victoria, Australia)

Krishna Mandaleson (Central Gippsland Health Service)

Romesh Markus (St Vincent's Hospital, Sydney, New South Wales, Australia)

Stephen Moore (Lismore Base Hospital, New South Wales, Australia)

Neha Nandal (Mackay Base Hospital, Queensland, Australia)

Kim Parrey (Port Macquarie Hospital, New South Wales, Australia)

Lauren Pesavento (Melbourne Health, Victoria, Australia)

Juan Rois-Gnecco (Ipswich, Queensland, Australia)

Darshan Shah (Gold Coast University Hospital, Queensland, Australia)

Daniel Schweitzer (Mater Hospital Brisbane, Queensland, Australia)

Amanda Siller (Queen Elizabeth II Jubilee Hospital, Queensland, Australia)

Jenni Steel (Port Macquarie Hospital, New South Wales, Australia)

Karen Stephens (Eastern Health, Victoria, Australia)

Louise Starkie (Hamilton Base Hospital, Victoria, Australia)

Meng Tan (Gold Coast University Hospital and Robina Hospital, Queensland, Australia)

Vincent Thijs (Austin Health, Victoria, Australia)

Dinesh Tryambake (Launceston General Hospital, Tasmania, Australia)

Rebecca Weir (Northeast Health Wangaratta, Victoria, Australia)

Leanne Whiley (Rockhampton Hospital, Queensland, Australia)

Richard White (Townsville Hospital, Queensland, Australia)

Tissa Wijeratne (Western Health, Victoria, Australia)

Matt Willcourt (Flinders Medical Centre, South Australia, Australia)

Nigel Wolfe (Blacktown Hospital, New South Wales, Australia)

Andrew Wong (Royal Brisbane and Women's Hospital, University of Queensland, Queensland, Australia)

Peter Wood (Bundaberg Hospital & Hervey Bay Hospital, Queensland, Australia)

Jorge Zavala (Alfred Health, Victoria, Australia).

## Conflict of Interest

The authors declare that the research was conducted in the absence of any commercial or financial relationships that could be construed as a potential conflict of interest.
