# Supplementary material for: COVID-19 Pandemic Impact on Care for Stroke in Australia: Emerging Evidence From the Australian Stroke Clinical Registry
Source: Front Neurol. 2021 Feb 26;12:621495. doi: 10.3389/fneur.2021.621495 (PMC7952624; doi:10.3389/fneur.2021.621495)
Supplement: Supplementary file 1 [file Data_Sheet_1.PDF]

Supplementary Material

COVID-19 Pandemic impact on Care for Stroke in Australia: emerging evidence from the Australian Stroke Clinical Registry

Dominique A. Cadilhac[1,2*], Joosup Kim[1,2], Emma K. Tod[2], Julie L. Morrison[2], Sibilah J. Breen[2], Katherine Jaques[3], Rohan Grimley[1,3], Brett Jones[4], Geoffrey Cloud[5,6], Timothy Kleinig[7], Susan Hillier[8], Helen Castley[9], Richard I. Lindley[10], Natasha A, Lannin[5,6], Sandy Middleton[11,12], Bernard Yan[13], Kelvin Hill[14], Benjamin Clissold[1,15], Peter Mitchell[13], Craig Anderson[16], Steven Faux[12,17], Bruce Campbell[13,2]

On behalf of the AuSCR COVID-19 reporting Consortium group*

1. Stroke and Ageing Research School of Clinical Sciences at Monash Health, Monash University, Clayton, Australia
2. Stroke Division, The Florey Institute of Neuroscience and Mental Health, Heidelberg, Australia
3. Stroke Statewide Clinical Network, Healthcare Improvement Unit, Clinical Excellence, Queensland Health, Brisbane, Australia
4. Canberra Hospital, Australian Capital Territory, Australia
5. Department of Clinical Neuroscience, Central Clinical School, Monash University, Clayton, Australia
6. Alfred Health, Prahran, Victoria, Australia
7. Royal Adelaide Hospital, Adelaide, South Australia
8. University of South Australia, Adelaide, South Australia
9. Royal Hobart Hospital, Hobart, Tasmania, Australia
10. University of Sydney, New South Wales, Australia
11. Australian Catholic University, Sydney, Australia
12. St Vincent's Health Network, Sydney, Australia
13. Department of Medicine and Neurology, Melbourne Brain Centre at the Royal Melbourne Hospital, University of Melbourne, Parkville, Victoria, Australia
14. Stroke Foundation, Melbourne, Victoria, Australia
15. Monash Health, Clayton, Victoria, Australia
16. The George Institute for Global Health, University of New South Wales, Sydney, New South Wales, Australia
17. University of New South Wales, Australia

***Correspondence:**
Dominique A. Cadilhac: dominique.cadilhac@monash.edu

Keywords: stroke, COVID-19, healthcare quality, survey, clinical registry

1 Supplementary Information

1.1 Methods for the design of the COVID-19 impact survey of hospitals involved in the Australian Stroke Clinical Registry

In total there were 23 questions included in the survey: clinician details (name, hospital and role), impact on role and ability to enter data into the registry, impacts on patient characteristics (number and type of presentations and time to presentation), and impact on stroke service delivery (e.g. time to brain scan or treatment, transfer protocols, changes in stroke unit size or staffing, length of stay and discharge destination). Most questions were optional with yes/no response options and free text boxes to provide further details. Free text boxes were also provided to allow respondents to detail changes not captured elsewhere and changes that had the greatest impact on stroke service delivery. The survey was designed to be completed electronically using Qualtrics software (version May 2020, Provo, Utah, United States of America, https://www.qualtrics.com).

The survey was distributed to the AuSCR Hospital mailing list and via an electronic Newsletter on the first of May 2020 and was left open until the 31st of May 2020. More than one response from each participating hospital was acceptable. Participation was voluntary.

1.2 Australian Stroke Clinical Registry (AuSCR) COVID-19 Impact Questionnaire for clinicians

**1. Name: Optional**		
**2. Hospital**		
**3. State**		
**4. Hospital role / position (e.g. stroke coordinator):**		
**AuSCR processes**		
5. **Has your hospital's capacity to enter AuSCR data been impacted?**	Yes	No
(*if Yes*): *Briefly describe how your capacity to enter AuSCR data has changed*	*Open text box*	
6. **Has your role at the hospital changed?**	Yes	No
(if Yes): Briefly describe how your role has changed	*Open text box*	

**Patient Characteristics**			
**Please indicate any impacts you have noted on the following:**			
**7. Total number of stroke presentations**	Lower	Same	Higher
**8. Number of TIAs and minor strokes**	Lower	Same	Higher
**9. Time from stroke onset to hospital presentation**	Shorter	Same	Longer

**Stroke Service Delivery**		
**Please indicate if any of the following aspects of stroke service delivery have changed at your hospital as a result of *COVID-19***		
**10. Time to brain scan**	Yes	No
(If Yes) Briefly describe the change	*Open text box*	
11. **Access to reperfusion therapies**	Yes	No
(If Yes) Briefly describe the change in access and if there has been an impact on stroke care	*Open text box*	
12. **Time to reperfusion therapy**	Yes	
(If Yes) Briefly describe the change	*Open text box*	
13. **Transfer protocols between hospitals**	Yes	No
(If Yes) Briefly describe the change in access and if there has been an impact on stroke care	*Open text box*	
**14. Other changes to patient flow/management in the Emergency Department.** *For example: processes relating to triage, diagnosis and contacting the stroke team or accessing Stroke Unit beds*	Yes	No
(If Yes) Briefly describe the changes to patient flow	*Open text box*	

15. **Stroke unit location**	Yes	No
(If Yes) Briefly describe the location change. Is the new location geographically close to/co-located with the previous location?	*Open text box*	
16. **Stroke unit size**	Yes	No
(If Yes) Briefly describe the change in stroke unit capacity	*Open text box*	
17. **Re-deployment of stroke staff to other duties (who, when and for what purpose)**	Yes	No
(If Yes) Briefly describe the staff re-deployment	*Open text box*	
18. **Length of acute care stay**	Yes	No
(If Yes) Briefly describe the change	*Open text box*	
19. **Discharge destination**	Yes	No
(If Yes) Briefly describe the change	*Open text box*	
**20. Please describe any other aspects of stroke service delivery that have been impacted.**	*Open text box*	
**21. Which of the changes in your stroke service at your hospital do you feel has had the greatest impact on the quality of stroke care?**	*Open text box*	
**Collection of COVID-19 impacts on individual patients**		
**22. Do you plan to, or are you currently, documenting delays in treatment or guideline adherence, due to hospital COVID-19 procedures for individual patients, (including those who are COVID-negative).**	Yes	No
**23. Are you interested in documenting the impacts on stroke care which may have occurred for individual COVID-19 positive patients in the AuSCR open text 'comments' box?**	Yes	No

2 Supplementary Figures and Tables

2.1 Supplementary Tables

Supplementary Table 1. Number of COVID-19 cases and stroke cases by State and Territory for the 61 hospitals contributing data in both periods

	Pandemic period		Control period
	**Number of confirmed COVID-19 cases in Australia***	**Number of stroke episodes registered in the AuSCR January-June 2020**	**Number of stroke episodes registered in the AuSCR January-June 2019**
New South Wales	3378	682	1080
Victoria	2159	4244	4141
Queensland	1067	2910	2850
South Australia	443	786	846
Western Australia	599	0	0
Tasmania	228	370	391
Northern Territory	29	0	0
Australian Capital Territory	108	0	0

* https://www.covid19data.com.au/ accessed 21 September 2020, AuSCR: Australian Stroke Clinical Registry (data as downloaded 17 September 2020)

Supplementary Table 2. Comparison of patient characteristics and arrival information for the historical control and national pandemic period

	Control period	Pandemic period	
	**January to June 2019**	**January to June 2020**	
Number of episodes	9,308	8,992	
Number of patients	9,116	8,729	
	**n (%)***	**n (%)***	**p-value**
Median age (IQR)	75 (64 - 83)	75 (65 - 83)	0.737
Male	5102 (56)	4953 (56)	0.999
Stroke type			0.533
Intracerebral hemorrhage	1124 (12)	1007 (12)	
Ischemic stroke	6393 (69)	5905 (69)	
Transient ischemic attack	1521 (16)	1475 (17)	
Undetermined stroke type	201 (2)	184 (2)	
Ability to walk on admission	3772 (43)	3159 (42)	0.167
Arrival by ambulance	6802 (77)	6127 (79)	<0.001
Median minutes from onset to arrival (IQR)	337 (100 - 1127)	348 (102 - 1096)	0.948

IQR: interquartile range; *number and proportion of the number of episodes unless otherwise specified

Supplementary Table 3. Interrupted times series analysis with comparison of trends in care access in Victoria and Queensland

Victoria	Trend before 1/3/2020	Change during the week of 1/3/2020 (interruption week)*	Trend from 1/3/2020
Proportion provided thrombolysis if an ischemic stroke	-0.07 (-0.09, -0.05)	1.90 (-0.09, 3.88)	-0.28 (-0.50, -0.06)
Median door to needle time (within 60 minutes)	0.24 (0.09, 0.40)	10.15 (4.85, 15.45)	-2.28 (-2.78, -1.79)
Proportion treated in a stroke unit	0.03 (-0.05, 0.10)	-5.63 (-8.84, -2.42)	-0.06 (-0.47, 0.35)
**Queensland**			
Proportion provided thrombolysis if an ischemic stroke	0.02 (-0.04, 0.08)	1.78 (-0.24, 3.80)	-0.26 (-0.32, -0.20)
Median door to needle time (within 60 minutes)	0.29 (0.13, 0.44)	6.50 (1.42, 11.57)	-1.96 (-2.46, -1.46)
Proportion treated in a stroke unit	-0.08 (-0.11, -0.05)	4.04 (2.70, 5.37)	-0.09 (-0.18, 0.003)

*represents week 61 of the interrupted time-series model with trends compared before and after this date, which coincides with the first COVID-19 related death

Supplementary Figure 1. Number of episodes entered into the Australian Stroke Clinical Registry per week

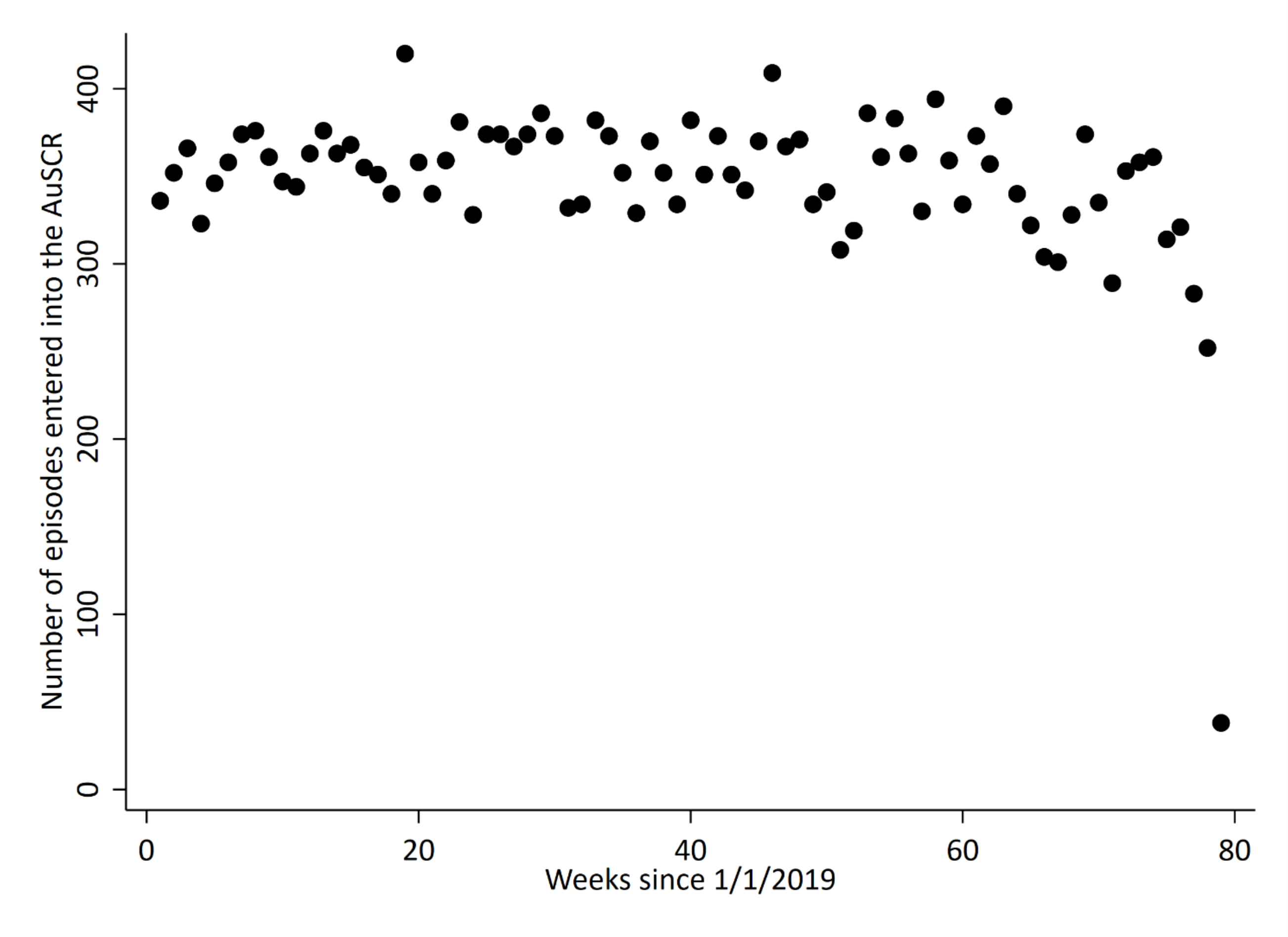

Supplementary Figure 2. Changes to hospitals as a result of COVID-19 reported by clinicians

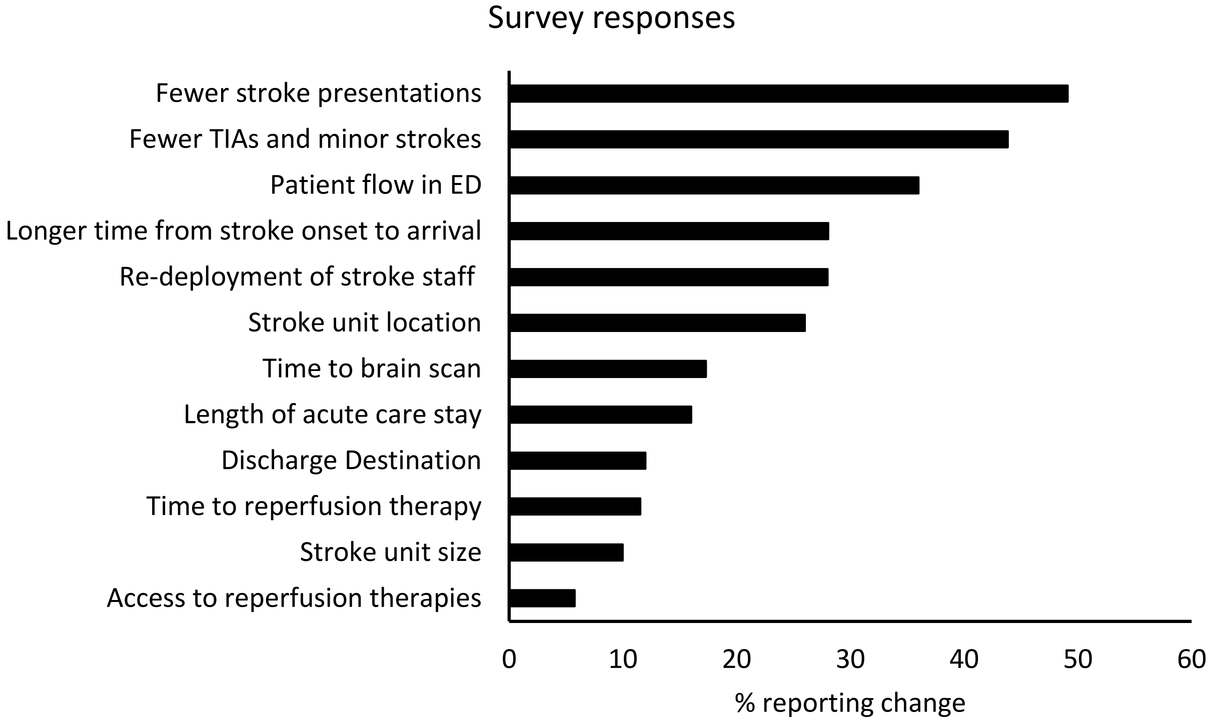

3. Supplemental acknowledgments

3.1 AuSCR data contributors not listed elsewhere

Evelyn Anino (Queen Elizabeth II Jubilee Hospital, Queensland, Australia)
Joanne Ashford (Mackay Hospital, Queensland, Australia)
Jennifer Bergqvist (Western Health, Victoria, Australia)
Haylee Berrill (Gold Coast University & Robina Hospitals, Queensland, Australia)
Pauline Blaney (Hervey Bay Hospital, Queensland, Australia)
Deirdre Broadby (Royal Hobart Hospital, Tasmania, Australia)
Michelle Bronca (Flinders Medical Centre, South Australia, Australia)
Camelia Burdusel (Blacktown Hospital, New South Wales, Australia)
Carla Borg Caruana (Alfred Health, Victoria, Australia)
Aylissa Canning (Princess Alexandra Hospital, Queensland, Australia)
Kristine Caprecho (Calvary Hospital, Australian Capital Territory, Australia)
John Carmody (Wollongong Hospital, New South Wales, Australia)
Shaji Chacko (Gympie Hospital, Queensland, Australia)
Kerri Chamberlain (Wimmera Base Hospital, Victoria, Australia)
Michelle Coad (Port Macquarie Hospital, New South Wales, Australia)
Kathryn Colebourne (The Prince Charles Hospital, Queensland, Australia)
Tessa Coupland (Bendigo Health, Victoria, Australia)
Shahla Cowans (Canberra Health Services, Australian Capital Territory, Australia)
Dijana Cukanovic-Krebs (Cairns Hospital, Queensland, Australia)
Sonia Dann (Bundaberg Hospital, Queensland, Australia)
Lizzie Dodd (Royal Adelaide Hospital, South Australia, Australia)
Helen Eaves (Bundaberg Hospital, Queensland, Australia)
Linda Edwards (Ipswich Hospital, Queensland, Australia)
Troy Elliott (Cairns Hospital, Queensland, Australia)
Tanya Frost (Eastern Health, Victoria, Australia)
Karen Fuller (Wollongong Hospital, New South Wales, Australia)
Renae Gamble (Austin Health, Victoria, Australia)
Belinda Green (Alfred Health, Victoria, Australia)
Patrick Groot (South West Healthcare, Victoria, Australia)
Kushantha Gunarathne (Barinsdale Regional health Service, Victoria, Australia)
Casey Hair (Ballarat Base Services, Victoria, Australia
Nicola Hall (Logan Hospital, Queensland, Australia)
Emma Harding (Swan Hill District Health, Victoria, Australia)
Carolyn Harrison (Launceston General Hospital, Tasmania, Australia)
Kim Hoffman (Lismore Base Hospital, New South Wales, Australia
Marnie Hollywood (Caboolture Hospital, Queensland, Australia)
Deborah Holmes (St Vincent's Hospital, Sydney, New South Wales, Australia)
Anne Hooper (Mackay Hospital, Queensland, Australia)
Tracey Howlett (Logan Hospital, Queensland, Australia)
Karen Hudson (Gympie Hospital, Queensland, Australia)
Michelle Hutchinson (Flinders Medical Centre, South Australia, Australia)
Manju John (Canberra Health Services, Australian Capital Territory, Australia)
Cath Jones (Bass Coast Health, Victoria, Australia)
Tracey Johnson (Gold Coast University Hospital, Queensland, Australia)

Caitlin Kearney (The Prince Charles Hospital, Queensland, Australia)
Lorraine Keene (West Gippsland Hospital, Victoria, Australia)
Kanaga Lagma (Peninsula Health, Victoria, Australia)
Kylie Lodge (Queen Elizabeth II Jubilee Hospital, Queensland, Australia)
Jodi Lynch (Monash Health, Victoria, Australia)
Laura Macdonald (Bairnsdale Regional health Service, Victoria, Australia)
Elizabeth Mackey (Western Health, Victoria, Australia)
Lyn Malone (Northeast Health Wangaratta, Victoria, Australia)
Janet May (Latrobe Regional Hospital, Victoria, Australia)
Marie McCaig (Mater Hospital, Queensland, Australia)
Amanda McKee (Princess Alexandra Hospital, Queensland, Australia)
Ian Meade (Townsville Hospital, Queensland, Australia)
Jessica Miller (Monash Health, Victoria, Australia)
Katherine Mohr (Wagga Wagga Hospital, New South Wales, Australia)
Maxine Munting (North West Regional Hospital, Tasmania, Australia)
Ashley Murray (West Gippsland Hospital, Victoria, Australia)
Linda Norrie (Townsville Hospital, Queensland, Australia)
Trish Oxley (Swan Hill District Health, Victoria, Australia)
Kirsty Page (St Vincent’s Hospital, Sydney, New South Wales, Australia)
Arun Raju (Toowoomba Hospital, Queensland, Australia)
Emara Ramchander (Western Health, Victoria, Australia)
Erin Ray (Bendigo Health, Victoria, Australia)
Deidre Rennick (Wimmera Base Hospital, Victoria, Australia)
Timothy Richardson (Toowoomba Hospital, Queensland, Australia)
Nina Roberts (Wimmera Base Hospital, Victoria, Australia)
Ros Roberts (Mildura Base Public Hospital, Victoria, Australia)
Anne Rodda (Northern Health, Victoria, Australia)
Simone Rogers (Bundaberg Hospital, Queensland, Australia)
David Rosaia (Bendigo Health, Victoria, Australia)
Donna Rowley (Sunshine Coast University Hospital, Queensland, Australia)
Ramesh Sahathevan (Ballarat Base Services, Victoria, Australia)
Sebastian Saj (Nepean Hospital, New South Wales, Australia)
Lauren Sanders (St Vincent’s Hospital, Victoria, Australia)
Mark Savage (Bendigo Health, Victoria, Australia)
Patrick Scarff (St Vincent’s Hospital, Victoria, Australia)
Betsy Shaju (Ipswich Hospital, Queensland, Australia)
Heather Smith (University Hospital Geelong, Victoria, Australia)
Jennifer Steel (Port Macquarie Hospital, New South Wales, Australia)
Margaret Stevenson (Peninsula Health, Victoria, Australia)
Leanne Taylor (Wimmera Base Hospital, Victoria, Australia)
Smisha Thomas (Royal Melbourne Hospital, Victoria, Australia)
Anne Van Berkel (Central Gippsland Health Service, Victoria, Australia)
Tamara Wanklyn (Western Health, Victoria, Australia)
Jaqueline Watson (Westmead Hospital, New South Wales, Australia)
Michelle Webb (Royal Brisbane and Women’s Hospital, Queensland, Australia)
Tracey Whitehead (Townsville Hospital, Queensland, Australia)
Tanya Williams (Redcliffe Hospital, Queensland, Australia)

Jerry Wong (Queen Elizabeth II Jubilee Hospital, Queensland, Australia)
Toni Wonson (Wollongong Hospital, New South Wales, Australia)
Melissa Wood (Royal Brisbane and Women's Hospital, Queensland, Australia)
Martin Zolfel (Nepean Hospital, New South Wales, Australia)

3.2 AuSCR Office Staff (not listed elsewhere)

Helen Carter (The Florey Institute of Neuroscience and Mental Health, Victoria, Australia)
Adele Gibbs (The Florey Institute of Neuroscience and Mental Health, Victoria, Australia)
Shaun Hancock (The Florey Institute of Neuroscience and Mental Health, Victoria, Australia)
Marcus Lester (The Florey Institute of Neuroscience and Mental Health, Victoria, Australia)
Violet Marion (The Florey Institute of Neuroscience and Mental Health, Victoria, Australia)
Karen Moss (The Florey Institute of Neuroscience and Mental Health, Victoria, Australia)
Kate Paice (The Florey Institute of Neuroscience and Mental Health, Victoria, Australia)
Nancy Pompeani (The Florey Institute of Neuroscience and Mental Health, Victoria, Australia)
Olivia Ryan (The Florey Institute of Neuroscience and Mental Health, Victoria, Australia)
Sam Shehata (The Florey Institute of Neuroscience and Mental Health, Victoria, Australia)
Claire Weickhardt (The Florey Institute of Neuroscience and Mental Health, Victoria, Australia)

3.3 AuSCR Committee Members (not listed elsewhere)

Julie Bernhardt (The Florey Institute of Neuroscience and Mental Health, Victoria, Australia)
Chris Bladin (The Florey Institute of Neuroscience and Mental Health, Victoria, Australia)
Ken Butcher (Prince of Wales Hospital, New South Wales, Australia)
Andrew Cheung (Prince of Wales and Liverpool Hospitals, New South Wales, Australia)
Philip Choi (Eastern Health, Victoria, Australia)
Alan Coulthard (Royal Brisbane and Womens Hospital, Queensland, Australia)
Coralie English (Hunter Medical Research Institute, New South Wales, Australia)
Sue Evans (Monash University, Victoria, Australia)
Darshan Ghia (Fiona Stanley Hospital, Western Australia, Australia)
Erin Godecke (Edith Cowan University, Western Australia, Australia)
Martin Krause (Royal North Shore Hospital, New South Wales, Australia)
Peter Hand (Alfred Health, Victoria, Australia)
Kate Jaques (Queensland Health, Queensland, Australia)
Suzanne Kuys (Australian Catholic University, Queensland, Australia)
Mark Mackay (Royal Children's Hospital, Victoria, Australia)
John McNeil (Monash University, Victoria, Australia)
Ferdi Miteff (Royal North Shore Hospital, New South Wales, Australia)
Jennifer Muller (Consumer representative, Queensland, Australia)
Lisa Murphy (Stroke Foundation, Melbourne, Victoria, Australia)
Michael Pollack (Hunter Stroke Service, New South Wales, Australia)
Rebecca Scroop (Royal Adelaide Hospital, South Australia, Australia)
Mark Simcocks (Consumer representative, Victoria, Australia)
Frances Simmonds (University of Wollongong, New South Wales, Australia)
Brendan Steinfort (Royal North Shore Hospital, New South Wales, Australia)
Belinda Stojanovski (Royal Children's Hosptial, Parkville
Amanda Thrift (Monash University, Victoria, Australia)

Jason Wenderoth (Prince of Wales Hospital, New South Wales, Australia)
Andrew Wesseldine (St John of God Hospital, Western Australia, Australia)
